# Single-Stranded Oligonucleotide-Mediated Inhibition of Respiratory Syncytial Virus Infection

**DOI:** 10.3389/fimmu.2020.580547

**Published:** 2020-12-08

**Authors:** Sandra Axberg Pålsson, Aleksandra Dondalska, Joseph Bergenstråhle, Caroline Rolfes, Albin Björk, Laura Sedano, Ultan F. Power, Marie-Anne Rameix-Welti, Joakim Lundeberg, Marie Wahren-Herlenius, Peter Mastrangelo, Jean-Francois Eleouet, Ronan Le Goffic, Marie Galloux, Anna-Lena Spetz

**Affiliations:** ^1^ Department of Molecular Biosciences, The Wenner-Gren Institute, Stockholm University, Stockholm, Sweden; ^2^ Science for Life Laboratory, Department of Gene Technology, Royal Institute of Technology, Stockholm, Sweden; ^3^ Division of Rheumatology, Department of Medicine, Karolinska Institutet, Karolinska University Hospital, Stockholm, Sweden; ^4^ UR0892 Unité VIM, INRAE, Université Paris-Saclay, Jouy-en-Josas, France; ^5^ Wellcome-Wolfson Institute for Experimental Medicine, School of Medicine, Dentistry and Biomedical Sciences, Queen’s University Belfast, Belfast, Northern Ireland; ^6^ UMR INSERM U1173 I2, UFR des Sciences de la Santé Simone Veil—UVSQ, Montigny-Le-Bretonneux, France; ^7^ Department of Laboratory Medicine and Pathobiology, University of Toronto, Toronto, Canada

**Keywords:** respiratory syncytial virus (RSV), oligonucleotides, single-stranded oligonucleotides, ssON, antiviral, nucleolin, ISGs

## Abstract

Respiratory syncytial virus (RSV) is the leading cause of acute lower respiratory tract infections in young children. Currently, there is no RSV vaccine or universally accessible antiviral treatment available. Addressing the urgent need for new antiviral agents, we have investigated the capacity of a non-coding single-stranded oligonucleotide (ssON) to inhibit RSV infection. By utilizing a GFP-expressing RSV, we demonstrate that the ssON significantly reduced the proportion of RSV infected A549 cells (lung epithelial cells). Furthermore, we show that ssON’s antiviral activity was length dependent and that both RNA and DNA of this class of oligonucleotides have antiviral activity. We reveal that ssON inhibited RSV infection by competing with the virus for binding to the cellular receptor nucleolin *in vitro*. Additionally, using a recombinant RSV that expresses luciferase we show that ssON effectively blocked RSV infection in mice. Treatment with ssON *in vivo* resulted in the upregulation of RSV-induced interferon stimulated genes (ISGs) such as *Stat1*, *Stat2*, *Cxcl10*, and *Ccl2.* This study highlights the possibility of using oligonucleotides as therapeutic agents against RSV infection. We demonstrate that the mechanism of action of ssON is the inhibition of viral entry *in vitro*, likely through the binding of the receptor, nucleolin and that ssON treatment against RSV infection *in vivo* additionally results in the upregulation of ISGs.

## Introduction

Human respiratory syncytial virus (RSV) is a negative sense single-stranded RNA virus of the *Pneumoviridae* family. It causes acute severe infections in the lower respiratory tract of children, the elderly and immunocompromised individuals (1). Annually, 64 million cases of RSV infection are reported globally and of these, approximately 160,000 cases result in death (National Institute of Allergy and Infectious Diseases; https://www.niaid.nih.gov). Furthermore, reports indicate that 90% of children are infected with RSV within their first two years of life (2). In addition, the development of wheezing and asthma have been associated with early-life RSV infection (3).

There is currently no RSV-specific antiviral treatment or vaccine against RSV, except for an immunoprophylactic anti-RSV monoclonal antibody (Palivizumab) or intravenous immunoglobulin (IVIG) treatment. Unfortunately, due to high cost, use of Palivizumab is restricted to children at a high risk of developing severe RSV-induced disease, such as premature infants, immunocompromised newborn babies, or children with congenital heart or lung disease, in high income countries (4, 5). Although several ongoing clinical studies are exploring potential RSV vaccines and new anti-RSV monoclonal antibodies, successful progress has been slow. In parallel, many recent studies are focusing on the development of alternative RSV inhibitors and the pipeline of new anti-RSV therapeutics was recently reviewed (6). Examples of new antiviral compounds are the fusion inhibitors Presatovir (GS-5806), TMC353121 and JNJ-678, and replication inhibitors such as Lumicitabine (ALS-8176) (7).

RSV was shown to enter ciliated epithelial cells *via* receptor-mediated endocytosis, utilizing nucleolin as a possible cellular receptor for viral entry (8). However, emerging studies indicate that RSV is taken up *via* macropinocytosis rather than endocytosis (9, 10). Hence, a detailed route of RSV infection remains to be further elucidated and entry is likely to involve a multimeric protein complex. Here, we investigated the prospect of using a phosphorothioate-stabilized 35 bases long single-stranded oligonucleotide (ssON) as an anti-RSV compound. The rationale was based on our previous findings showing that ssON inhibited clathrin- and caveolin-dependent endocytic uptake (11) as well as inhibited influenza A virus (IAV) infection *in vitro* and *in vivo* (12).

Infection of A549 cells (adenocarinoma-derived alveolar type II pneumocytes) with a RSV A virus expressing GFP and the quantification of infected cells using flow cytometry allowed us to generate evidence that ssON inhibits RSV infection in A549 cells *in vitro*. We show that ssON acts as an entry inhibitor by interfering with the cellular binding of RSV to nucleolin *in vitro*. Additionally, we present compelling *in vivo* data revealing that ssON treatment inhibits RSV infection in mice and moreover results in an increased expression of ISGs, which likely contributes to the antiviral effects obtained *in vivo*.

## Results

### Anti-Respiratory Syncytial Virus Activity of ssON *In Vitro*

**Figure 1:**
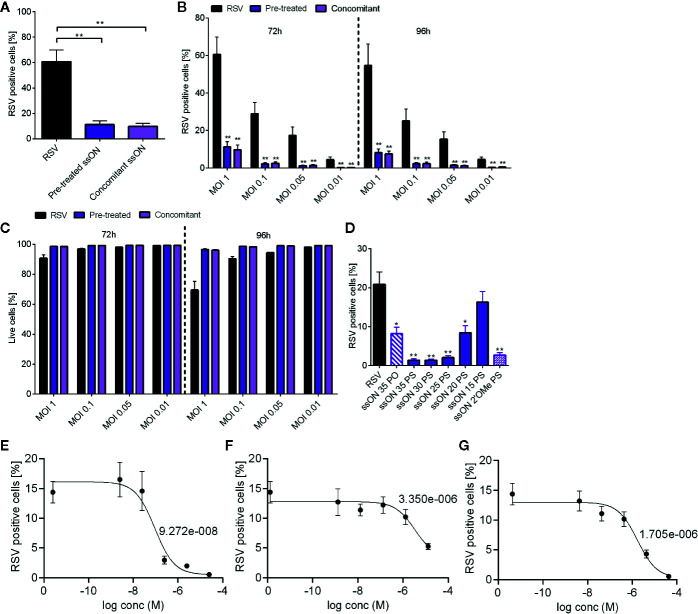
Anti-respiratory syncytial virus (RSV) activity of ssON *in vitro*. A549 cells were infected with a RSV expressing GFP (RSV-GFP), at the indicated multiplicities of infection (MOI) for 2 h. Cells were further incubated for indicated time points prior to staining with LIVE/DEAD^®^ Fixable near-IR Dead Cell Stain Kit **(A)**. Cells were either pre-treated with a 35 bases long PS-stabilized ssON (ssON) for 2h or ssON (1 µM) was added simultaneously with RSV (MOI 1). The proportion of GFP positive live cells was determined 72 h post infection using flow cytometry **(B)**. ssON was added to cells 2h before or concomitantly with RSV infection (MOI 1, 0.1, 0.05 or 0.01). The proportion of GFP positive live cells were measured 72 h and 96 h post infection using flow cytometry **(C)**. The viability of RSV and ssON treated cells was measured 72 h and 96 h post infection using flow cytometry **(D)**. Cells were treated with a 35 bases long un-stabilized ssON PO (ssON 35 PO) (1 µM) or PS-ssONs (1 µM) of different lengths as indicated by a number (ssON 30 PS etc). Alternatively, cells were treated with a 35 bases long single-stranded RNA with stabilizing modifications (ssON 2´OMe PS) 2 h before RSV infection at MOI 0.05. The proportion of live infected cells was measured 72 h post infection by flow cytometry. A549 cells were treated with **(E)** ssON **(F)**, TMC353121 or **(G)** Presatovir (GS-5806) at indicated concentrations for 2h prior to RSV infection (MOI 0.05). The proportion to live infected cells was measured 72h post infection by flow cytometry and the IC50s are depicted in the graphs. Results are presented as mean ±SEM from three independent experiments in duplicates for each time point for all graphs. The statistical significance was measured using the non-parametric Mann-Whitney test for A, B, and C; all treatments were compared to the RSV infected untreated control for statistics. For G, H, and I the statistical significance was measured using the Kruskal-Wallis one-way ANOVA test with Dunns multiple comparison between each treatment conditions and the untreated RSV infected cells (control). *P*-value: *P* > 0.05; *P ≤ 0.05; **P ≤ 0.01. Lack of significance is not depicted in the figure.

RSV is reported to enter cells *via* receptor-mediated endocytosis (8, 13, 14). We thus assessed if ssON, which was previously shown to inhibit clathrin- and caveolin-mediated endocytosis (11), could inhibit RSV infection. We evaluated anti-RSV activity against a RSV-A virus expressing GFP (RSV-GFP) by assessing the proportion of A549 infected cells using flow cytometry (**Supplementary Figure 1**, gating strategy). Pre-treatment with ssON or concomitant addition of ssON and RSV to A549 cells resulted in a significantly reduced proportion of GFP positive RSV-infected cells (~10%) (**Figure 1A**) as compared to the RSV-infected untreated controls (~60%).

We next evaluated ssON´s anti-RSV activity using MOIs ranging from 0.01 to 1 (**Figure 1B**). We found that ssON effectively blocked RSV infection for all MOIs assessed regardless of whether ssON was added before infection or simultaneously with the virus (**Figure 1B**). The antiviral activity persisted for up to 96h after a single ssON exposure. We also monitored cell viability and found that the highest RSV infection dose (MOI 1) resulted in increased cell death overtime (~10-30%). Notably, neither pre-incubation nor concomitant addition of ssON and RSV negatively affected the viability of A549 cells. Instead, there was a clear trend that ssON treatment reduced the cell death by up to 25% in the cell cultures exposed to the highest MOI 1 (**Figure 1C**).

We have previously reported that the capability of ssONs to interfere with endocytosis is dependent on the length of the oligonucleotide. Oligonucleotides with the same lengths as miRNAs do not possess this feature. However, somewhat longer oligonucleotides of at least 25 bases may exert such activity (11). The endocytosis inhibitory capacity is not based on complementary binding to another oligonucleotide but requires certain structural features to have this activity (11). In addition, there is extensive data in the literature that the chemical phosphorothioate (PS) modification of the backbone prolongs the half-life of the oligonucleotides both *in vitro* and *in vivo* as compared with naturally occurring oligonucleotides containing a phosphodiester (PO) backbone (12, 15). To evaluate whether the same structural features of ssONs required to block endocytosis are also needed to exert anti-RSV activity, we treated A549 cells with oligonucleotides of different lengths and chemical modifications prior to RSV infection. We found that a length of at least 20 bases was necessary for the ssONs to affect RSV infection, confirming a minimum length requirement for the effect (**Figure 1D**). Our results also showed that ssONs can be composed of either DNA (all ssONs marked as PS or PO) or RNA nucleotides (ssON 2’OMe PS) to effectively block RSV infection in A549 cells. These data are in accordance with our previous studies demonstrating some key structural features required to inhibit endocytosis (11). Moreover, we found that the PS stabilizing modification improved the efficiency of ssON to block RSV infection. However, the modification was not required as the naturally occurring ssON-PO could also inhibit the infection albeit with reduced efficiency as compared with the PS-ssON (**Figure 1D**).

ssON showed nanomolar activity (IC50 approximately 92.7nM) against RSV infection in the A549 cell assay (**Figure 1E**). This compared very favorably with other RSV entry inhibitors such as TMC353121 (IC50 3.35 µM) (**Figure 1F**), or Presatovir (IC50 1.7 µM) (**Figure 1G**), which were analyzed side-by-side. Taken together, these data confirm that ssON works efficiently as an antiviral agent against RSV in A549 cells.

### ssON Binds to Cell Surface Expressed Nucleolin *In Vitro*

**Figure 2:**
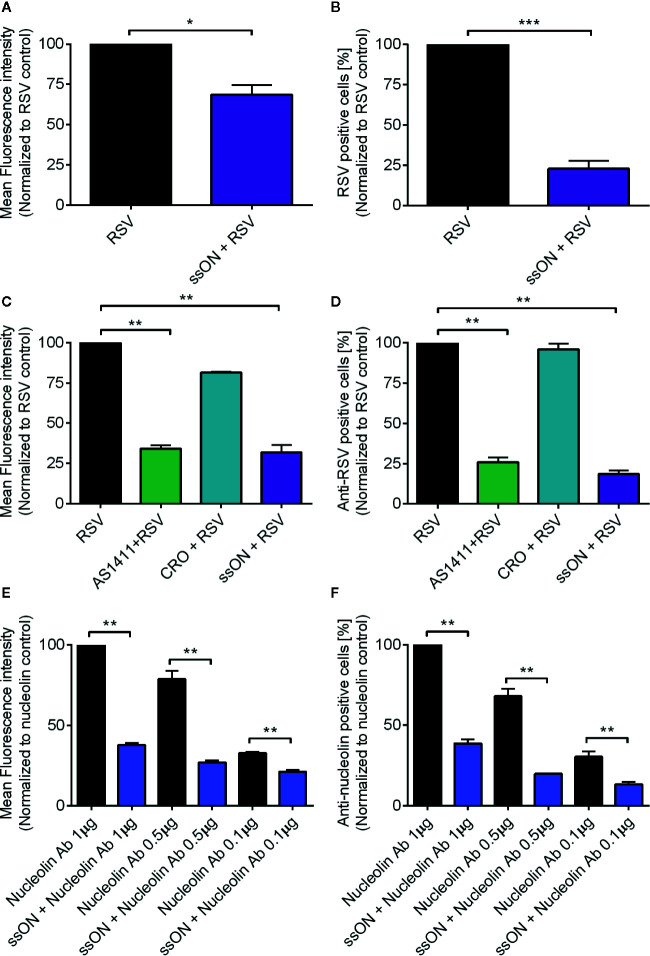
ssON binds to cell surface expressed nucleolin. A549 cells were treated with ssON (1 µM), AS1411 (25 µM), or CRO (25 µM) for 30 min prior to incubation with respiratory syncytial virus (RSV) (MOI 2) for 1h at 4°C followed by detection with an anti-RSV F protein mAb combined with a APC-conjugated rat anti-mouse IgG1 secondary antibody. Cell surface bound RSV was subsequently detected by flow cytometry **(A)**. Mean fluorescence intensity (MFI) of bound RSV in the presence or absence of ssON **(B)**. Proportion of RSV positive cells in the presence or absence of ssON **(C)**. MFI of cell surface bound RSV in the presence or absence of AS1411, CRO, or ssON **(D)**. Proportion of RSV positive cells in the presence or absence of AS1411, CRO, or ssON. The data was normalized to the untreated RSV control. Alternatively, cells were treated with ssON (1 µM) for 30 min prior to incubation with an anti-nucleolin antibody at the indicated concentrations for 1 h at 4°C followed by staining with an Alexa 488-conjugated donkey anti-rabbit IgG secondary antibody **(E)**. MFI of cell surface expressed nucleolin and **(F)** the proportion of cells labeled with an anti-nucleolin mAb was measured using flow cytometry. The data was normalized to the nucleolin 1 µg control. Results are presented as mean ±SEM from three to four independent experiments in duplicates for all graphs. The statistical significance for all graphs was measured using the non-parametric Mann-Whitney test and all treatments were compared to the RSV control. *P*-value: *P* > 0.05; *P ≤ 0.05; **P ≤ 0.01; ***P ≤ 0.001. Lack of significance is not depicted in the figure.

Next, we wanted to determine at what stage in the viral entry cycle ssON exerted the antiviral effect. We first assessed whether ssON interferes with extracellular binding of RSV using an established flow cytometry based cell-binding assay (9). We treated A549 cells with ssON 30 min prior to incubation with RSV. All treatments were performed at 4°C to ensure that no viral entry occurred. We used the RSV-GFP virus for the binding assay. However, the GFP protein is only expressed after viral replication inside host cells following infection. Therefore, we stained the extracellular virions with an anti-RSV F-protein antibody and the mean fluorescent intensity (MFI) and the proportion of RSV positive cells were measured using flow cytometry. We found that both the MFI (**Figure 2A**) and the proportion of RSV positive cells (**Figure 2B**), were significantly reduced in the presence of ssON, showing that fewer virions bound to the cells in the presence of ssON compared to the untreated cells. This clearly indicated that ssON inhibited RSV from binding to the cells.

RSV was reported to utilize nucleolin as a cellular receptor for entry (8). We therefore investigated whether nucleolin was required for RSV infection of A549 cells. For this purpose, we used a DNA aptamer (AS1411) that binds to nucleolin (16, 17) and thereby blocks RSV infection (18). We also used a cysteine-rich DNA aptamer that does not bind to nucleolin as a negative control (CRO) in the flow cytometry-based RSV binding assay (18). We found that RSV binding to A549 cells in the presence of AS1411 was diminished, as evidenced from a significant reduction in both the MFI (**Figure 2C**) and the proportion of RSV positive cells (**Figure 2D**) as compared to the untreated controls. Furthermore, the presence of CRO did not affect RSV binding, confirming previous reports showing that RSV binding to nucleolin facilitated infection of A549 cells.

Next, we investigated if ssON binds to nucleolin as a possible mechanism of action *in vitro*, explaining how it blocks RSV from binding to A549 cells. To investigate this, we used the flow cytometry-based cellular binding assay in combination with an anti-nucleolin antibody. We indeed revealed that pre-incubation with ssON resulted in reduced binding of the anti-nucleolin antibody, which could be measured for both the MFI (**Figure 2E**) and the proportion of anti-nucleolin positive cells (**Figure 2F**). We used several concentrations of the anti-nucleolin antibody and ssON was able to inhibit binding of the antibody, irrespective of the antibody concentration used, showing that ssON efficiently binds surface nucleolin on A549 cells. In summary, we show that both RSV and ssON bind to nucleolin expressed on A549 cells, suggesting that ssON prevents RSV infection by competing with the virus for nucleolin binding *in vitro*.

### Treatment With ssON Inhibits Respiratory Syncytial Virus Infection *In Vivo* in Mice

**Figure 3:**
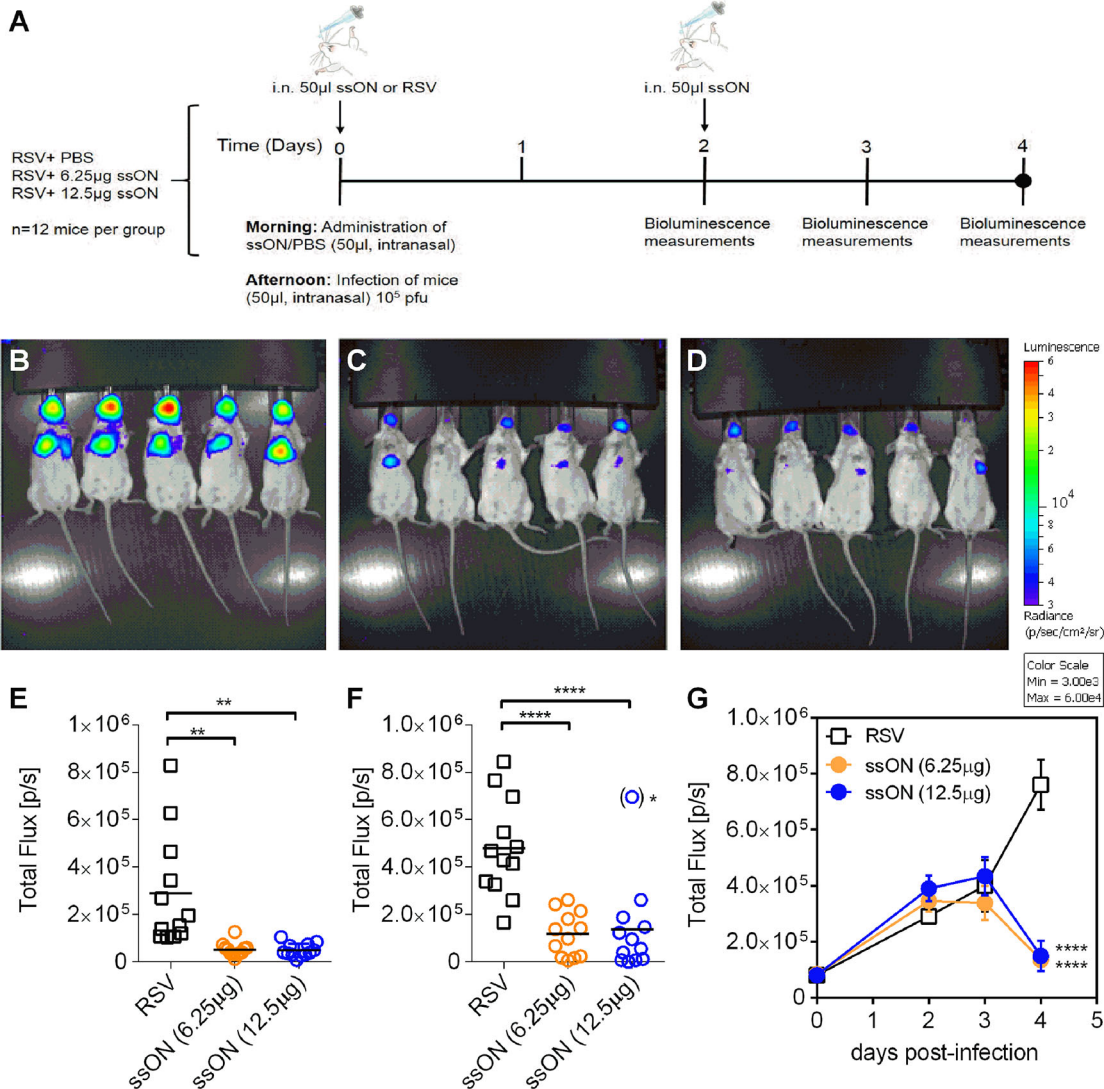
Treatment with ssON inhibits respiratory syncytial virus (RSV) infection *in vivo* in mice. Mice were infected intranasally with RSV-luciferase (2.10^6^ pfu/ml) according to the schedule depicted in **(A)**. Mice were given PBS or ssON (12.5 µg or 6.25 µg) intranasally 6 h before infection. A second treatment of ssON was administrated intranasally on day 2. The bioluminescence was measured using a *in vivo system* imaging (IVIS) after intranasal administration of D-luciferin, and capturing photon emission on day 0, 2, 3, and 4 post infection. Representative pictures of bioluminescence measured on the ventral view of mice after 4 days of infection with **(B)** RSV **(C)**, RSV + ssON (6.25 µg), and **(D)** RSV + ssON (12.5 µg). The scale indicates the radiance in photons per second per square centimeter per steradian (p/s/cm2/sr). The “Living Image” software was used to quantify the luciferase activity (expressed as photon per seconds) on day 4 in **(E)** nose and **(F)** lungs ()* = outlier which was excluded from the statistical analysis. **(G)**. The luciferase activity of the entire mouse was measured at indicated time points. Two independent experiments were performed and data show mean ±SEM with n=12 mice for each time point for all graphs. The statistical significance for all graphs was measured using an unpaired t-test with Welch’s correction comparing each treatment to the RSV control. *P*-value: *P* > 0.05; *P ≤ 0.05; **P ≤ 0.01; ****P ≤ 0.0001. Lack of significance is not depicted in the figure.

To investigate ssON’s effect *in vivo* we used a reporter virus (RSV-Luciferase) in combination with an *in vivo* imaging system (IVIS) to enable visualization and quantification of the replication as well as the spread of RSV in mice (19). Wild type BALB/c mice were treated with two different concentrations of ssON (12.5 µg/0.625 mg/kg or 6.25 µg/0.313 mg/kg) 6h prior to infection with RSV-Luciferase *via* intranasal administration. The ssON was administrated again 2 days post infection using the same doses. The initial infection and spread of RSV in the presence of ssON was determined daily by measuring the *in vivo* bioluminescence up to day four according to the schedule depicted in **Figure 3A**. The spread of RSV was detected in both the upper and lower respiratory tract of all mice 4 days post infection, as expected. Notably, the bioluminescence in the nose and lungs was significantly reduced in mice treated with both of the concentrations of ssON as compared to the untreated RSV infected mice (**Figures 3B–D**).

The quantification of the imaging established that ssON treatment, using a dose of 0.625 mg/kg or 0.313 mg/kg, resulted in significantly lower RSV levels four days post infection as compared to the RSV control. The anti-viral activity was observed both in the nose and in the lungs of mice (**Figures 3E, F**). One mouse treated with 12 µg ssON had elevated levels of RSV in the lungs compared to the other mice treated with same dose of ssON. The diminished treatment effect in this outlier could be due to sneezing, which occurs occasionally when compounds are administrated intranasally. The ssON treatment 6h prior to infection could not prevent the initial infection of the mice (**Figure 3G**). However, following the addition of a second dose of ssON on day two, there was a significant antiviral effect evident four days after infection. This indicates that ssON did not completely inhibit the initial inoculum of viruses (1x10^5^ pfu) *in vivo* but that the second dose of ssON was able to limit further spread of the virus.

### Treatment With ssON Against Respiratory Syncytial Virus Infection *In Vivo* in Mice Upregulates the Expression of Interferon Stimulated Genes

Pathology studies of the murine lung tissue following infection showed that neither the infection alone nor the infection in combination with ssON treatment had any gross pathological effect on the lungs when compared to the PBS treatment (**Supplementary Figure 2**).

We decided instead to assess ssON’s effect on the immune response in mice following RSV infection and therefore, we extracted RNA from lungs collected four days post infection. The immune gene profile of 547 immune genes was investigated using Nanostring analysis comparing non-infected mice, RSV-infected mice or RSV-infected mice treated with ssON. In this analysis 61 genes were defined as ISGs according to previous list by Barouch et al. (20) (see **Supplementary Table 1** for ISGs included in this study).

Infection alone and infection in combination with ssON treatment constituted the main driver of variance in the immune profile between the samples (**Supplementary Figures 3A, B**). Furthermore, for the genes contributing to most of the variance between the samples, treatment with ssON resulted in an even more altered expression compared to the change induced by infection alone, while also demonstrating higher intra-sample variability (**Supplementary Figure 3B**).

**Figure 4:**
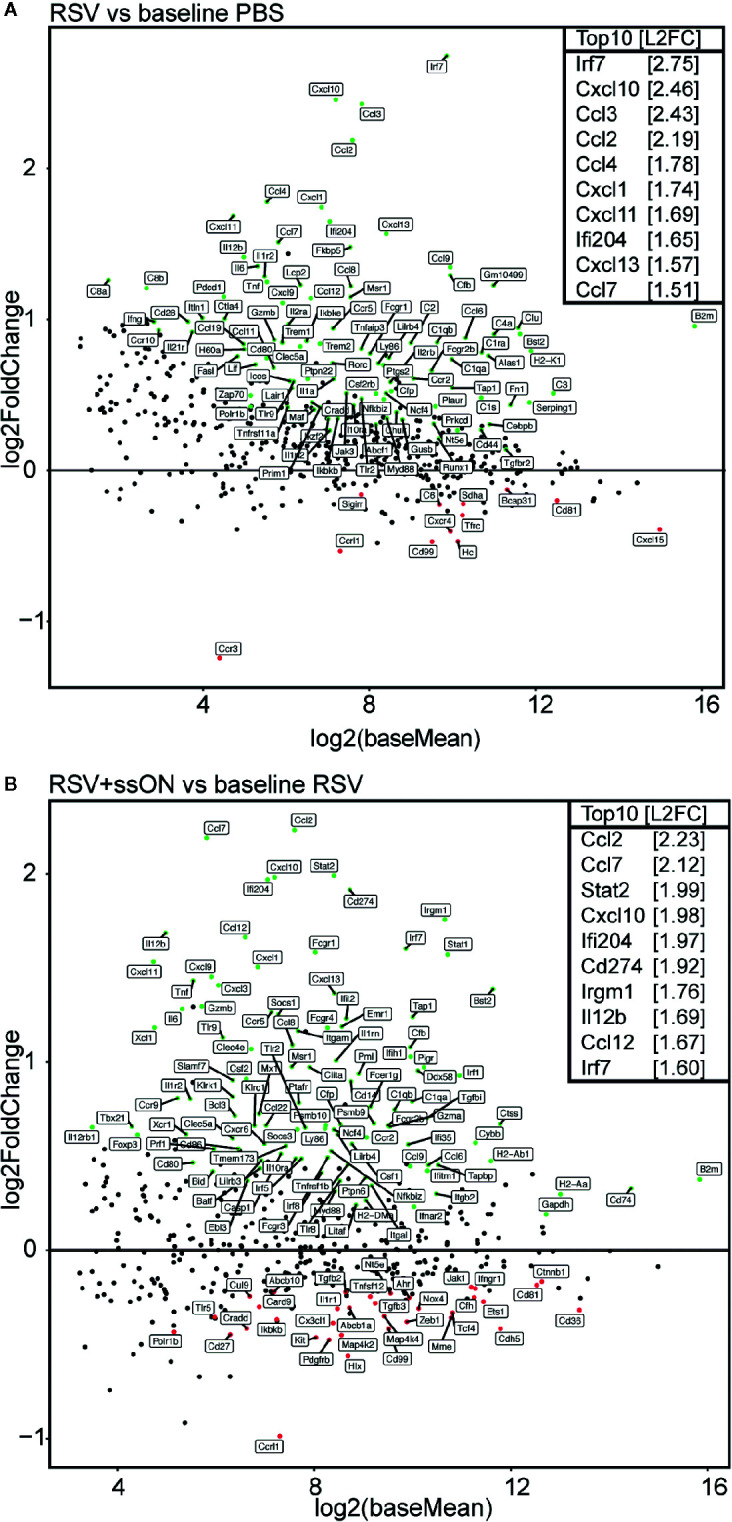
ssON treatment *in vivo* induces a differential immune profile in the lungs of respiratory syncytial virus (RSV) infected mice. RNA was extracted from the right lung lobes of mice treated with PBS, infected with RSV or from RSV infected mice treated with ssON (6.5 µg) 4 days post infection and analysed using Nanostring technology. Data consist of 9–10 mice per treatment from two independent experiments. DESeq2 analysis was used to normalize the immune gene expression to the internal reference genes. Genes with an adjusted p-value below 0.05 are fully colored (Significantly upregulated genes = green, significantly downregulated genes = red, rest = black) **(A)**. MA-plots showing the expression profile in log2 fold change (L2FC) of immune genes in RSV infected mice compared to PBS treated mice. The top 10 most upregulated genes by L2FC are displayed **(B)**. MA-plots showing the expression profile in L2FC of immune genes in ssON treated RSV infected mice compared to RSV infected mice. The top 10 most upregulated genes by L2FC are displayed.

RSV infection resulted in a significant upregulation of 102 immune genes and downregulation of 12 immune genes as compared to the immune gene profile of the non-infected control mice (**Figure 4A**, **Supplementary Figure 3C** and **4A**). Furthermore, the analysis showed that RSV infection induced the upregulation of the expression of ISGs. In total 20 ISGs were significantly upregulated in the RSV-infected mice compared to the non-infected mice (**Supplementary Table 1**) and some of the top 10 upregulated genes, such as *Irf7,Cxcl10*, *Ccl2*, and *Ccl4*, are all ISGs known to be induced in response to viral infections (21–24).

When comparing the immune gene profile of untreated RSV infected mice to ssON-treated RSV-infected mice, there was a significant upregulation of 109 immune genes and a downregulation of 35 immune genes in the ssON-treated mice compared to the untreated mice (**Figure 4B**, **Supplementary Figures 3C**, **4B**). Interestingly, ssON treatment of RSV-infected mice resulted in an increased upregulation of several RSV-induced ISGs. A total of 31 ISGs were upregulated in the ssON-treated RSV-infected mice compared to the untreated RSV-infected mice (**Supplementary Table 1**). For example, ssON treatment of RSV-infected mice led to an upregulation in expression of ISGs such as *Stat1*, *Stat2*, *Cxcl10*, and *Ccl2*. All of these ISGs play an important role in host defense against RSV (21, 25, 26). Furthermore, ssON treatment in the absence of RSV infection did not induce any altered expression of ISGs (**Supplementary Figure 5**), indicating that ssON treatment can enhance the RSV-induced expression of ISGs, but does not induce the expression of ISGs by itself.

**Figure 5:**
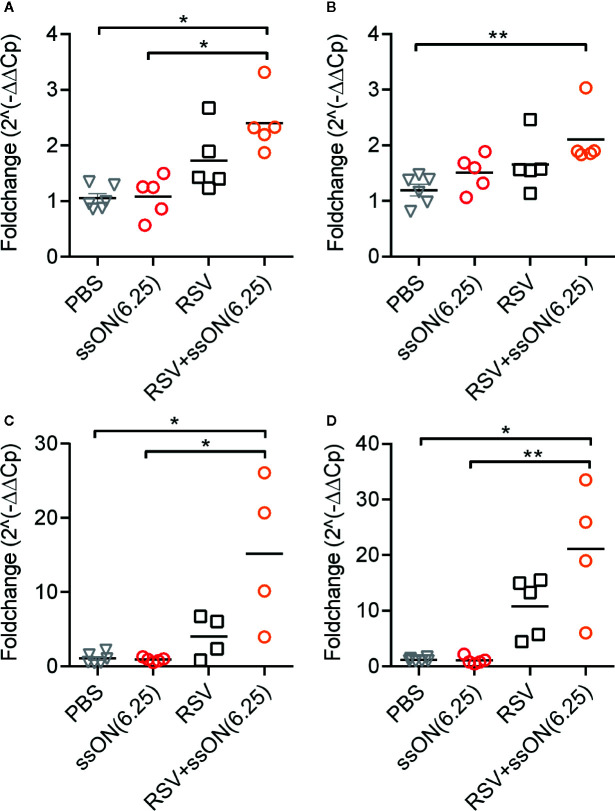
Treatment with ssON against respiratory syncytial virus (RSV) infection *in vivo* in mice upregulates the expression of interferon stimulated genes (ISGs). The expression of selected ISGs in the murine lungs was validated using RT-qPCR **(A)**. S*tat1* **(B)** S*tat2* **(C)** C*cl2* **(D)** C*xcl10*. Plots show mean from individual animals. Statistical significance was measured using the Kruskal-Wallis one-way ANOVA test with Dunns multiple comparison between each treatment conditions. *P*-value: *P* > 0.05; * *P* ≤ 0.05; ** *P* ≤ 0.01. Lack of significance is not depicted in the figures.

Validation of the Nanostring data using RT-qPCR confirmed that RSV infection induced upregulation of ISGs such as *Stat1* (**Figure 5A**), *Stat2* (**Figure 5B**), *Ccl2* (**Figure 5C**), and *Cxcl10* (**Figure 5D**) compared to the non-infected control. The RT-qPCR data also confirmed the upregulation of ISGs in RSV-infected mice treated with ssON as revealed in the Nanostring analysis. These results indicate that ssON treatment in combination with RSV infection induces increased expression of ISGs *in vivo*.

## Discussion

Herein, we present evidence that ssON inhibits RSV infection *in vitro* and *in vivo*. We show that ssON binds to nucleolin, a RSV receptor or entry factor, in A549 cells *in vitro*, thereby preventing the virus from binding to the cells. Furthermore, we show that ssON efficiently reduced RSV infection *in vivo* in BALB/c mice. Additionally, we provide evidence that ssON also functions in an immunomodulatory way *in vivo* in mice upon RSV infection. Our findings are of importance as there are no vaccines or antivirals against RSV currently available and therefore, there is a great need for new effective antiviral treatments.

We have reported in previous studies that ssON can inhibit certain endocytic pathways, such as the clathrin- and caveolin-dependent endocytosis (11). As RSV is reported to enter cells *via* receptor-mediated endocytosis, we sought to investigate if the endocytosis inhibitor ssON can inhibit viral entry into cells. We assessed ssON’s capacity as a RSV entry inhibitor in A549 cells by pre-treating the cells with ssON or adding ssON simultaneously with the virus and determined the proportion of RSV positive cells using flow cytometry. We found a significant reduction in the proportion of RSV infected cells in the presence of ssON. To investigate ssON’s efficiency as an RSV inhibitor, we conducted side-by side comparisons with other RSV entry inhibitors. We discovered that ssON had an IC50 of around 92.7 nM, while the IC50 in this assay was 3.35 µM for TMC353121 and 1.7 µM for Presatovir. Thus, our data suggest that ssON is approximately 36- and 18-fold more effective, respectively, than the latter drugs at inhibiting RSV infection *in vitro*.

We have previously demonstrated that ssON’s effect on endocytosis is length- but not sequence-dependent (11, 12). We confirmed here that ssON’s capacity to inhibit RSV is also size-dependent, as oligonucleotides of a length shorter than 20 bases did not have the ability to inhibit the infection. In previous studies the PS backbone was shown to confer all the antiviral effect of the oligonucleotide, while the natural PO backbone did not exhibit this effect (15, 27). However, we report here that the stabilizing PS modification of the backbone is not necessary for the inhibition of RSV infection, although it enhanced the efficiency of the inhibition compared to ssONs with the natural PO backbone. Furthermore, we show that ssONs composed of either DNA or RNA nucleotides have inhibitory effects on RSV infection, indicating that the type of nucleic acid is inconsequential to the ssONs antiviral effect. This intriguing finding also opens up the theoretical possibility that naturally occurring RNAs released into the extracellular space may exert this antiviral effect before their degradation.

Next, we demonstrated that ssON inhibited the cellular binding of the virus as determined by a flow cytometry based binding assay. Although some recent studies show that RSV might be taken up *via* macropinocytosis, most studies have reported that the virus can utilize receptor mediated-endocytic uptake, indicating nucleolin as a potential receptor (8). Indeed, a recent article confirmed nucleolin as an important co-receptor for RSV entry both *in vitro* and *in vivo* (28). Although, nucleolin is primarily located in the nucleolus of cells, it can also be present in the cytoplasm as well as the plasma membrane, where it is reported to interact with the RSV F protein, which facilitates the viral binding (29). Furthermore, RSV was shown to recruit nucleolin to the surface of cells thereby aiding its own uptake (28). Nucleolin contains RNA recognition motifs that have been reported to bind RNA, as well as single stranded DNA in some cases (30). Additionally, studies have indicated that oligonucleotides with a PS backbone can bind in a sequence-independent manner to Nucleolin (31), further supporting its role as an interaction partner of ssON. Indeed, we show here that both RSV and ssON bind to nucleolin in A549 cells. This suggests that pre-treatment with ssON inhibits RSV from binding to nucleolin and thereby prevents the virus from infecting cells. Nucleolin is implicated to take part in multicomplex interactions occurring during viral binding and uptake of a plethora of viruses, such as RSV, HIV-1 and HSV-2 (8, 28, 30). It remains to be further elucidated which viral families use nucleolin to enter target cells and whether ssON has the capacity to act as a broad-spectrum antiviral agent. Furthermore, as ssON has previously been shown to inhibit receptor-mediated endocytosis (11) it is reasonable to hypothesize that ssON’s mode of action *in vitro* does not solely encompass the prevention of RSV infection *via* interference with nucleolin binding, but may additionally affect other factors involved in RSV entry, thus supporting the observed efficiency of ssON as an RSV inhibitor *in vitro*.

Finally, we used a RSV reporter virus (RSV-luciferase) and an *in vivo* imaging system (IVIS) to investigate the effect of ssON on RSV infection in BALB/c mice (18). We demonstrated that ssON significantly reduced RSV infection and viral spread in the respiratory tract of mice. The initial ssON treatment given 6h prior to infection was not able to block RSV infection *in vivo*. The reason for the lack of prophylactic activity is most likely due to organ distribution and metabolism of ssON *in vivo*, which have been well documented for similar classes of ssONs (32). However, following a second administration of ssON on day two, we measured a significant and robust reduction of RSV infection in the nose and lungs of the mice at day four. More in-depth studies regarding treatment intervals would be required to optimize ssON’s therapeutic utility. Interestingly, Nanostring and RT-qPCR data showed that ssON treatment of RSV infected mice had an immunomodulatory effect on multiple RSV-induced ISGs, which would explain the reduced infection by day 4. We show that ssON treatment upregulated RSV induced expression of ISGs, such as *Stat1* and *Stat2*, which are important components of the Janus kinase/Signal Transducers and Activators of Transcription- (JAK/STAT) pathway responsible for inducing host antiviral defenses (25, 26). Furthermore, ssON treatment of RSV-infected mice increased the RSV-induced expression of *Cxcl10* and *Ccl2*, which are both reported to recruit immune cells, such as monocytes, dendritic cells and T cells, to sites of infection or inflammation, thereby aiding in viral clearance (21, 33, 34). It is conceivable that by preventing viral entry using ssON the virus is forced to enter cells through alternative pathways and this could result in the increased induction of certain ISGs. Alternatively, and not mutually exclusive, the prolonged stay of the virus in the extracellular space following lack of entry may allow for uptake in or responses from recruited immune cells resulting in the increased induction of ISGs. The finding that ssON treatment did not lead to induction of ISGs supports our hypothesis that it is the virus alone that induces the ISGs in ssON-treated RSV-infected mice. Evidently, further studies are required to confirm that ssON binding to nucleolin is the mechanism of antiviral action against RSV *in vivo*.

Altogether, our data demonstrate that ssON has significant potential as a novel potent antiviral agent against RSV. Its capacity to prevent RSV interaction with nucleolin *in vitro* and enhance ISG expression following RSV infection *in vivo* provide strong mechanistic rationales for the further development of ssON.

## Materials and Methods

### Cells and Virus

A549 lung epithelial cells were purchased from the American Type Culture Collection. The cells were cultured in DMEM high glucose (Hyclone) supplemented with 5% FBS (Sigma Aldrich), 1% PEST, 1% L-glutamine, 1% Hepes, and 1% sodium pyruvate. Cells were seeded 24h prior to infection at a concentration of 5x10^4^ cells/well in 24 well plates. RSV A expressing recombinant GFP (RSV-GFP) (35) and RSV-Luciferase 04/2017 were produced as previously described (19).

### Oligonucleotides and Inhibitors

A 35 bases long fully phosphorothioate-modified oligonucleotide, designated ssON, with sequence: 5’-GAAGTTTTGAGGTTTTGAAGTTGTTGGTGGTGGTG-3’, was purchased from Integrated DNA Technologies. The 2′OMe PS with sequence GAAGUUUUGAGGUUUUGAAGUUGUUGGUGGUGGUG was also purchased from Integrated DNA Technologies. The 30-mer (5′-AGTTTTGAGG TTTTGAAGTTGTTGGTGGTG-3′), the 25-mer: 5′-TTTGAGGTTTTGAAGTTGTTGGTGG-3′, the 20-mer: 5′-TGAGGTTTTGAAGTTATTGG-3′ and the 15-mer: 5′- GGTTTTGAAGTTGTT-3′, were all purchased from Eurofins. RSV fusion inhibitors TMC353121 and Presatovir (GS-5806) were purchased from MedChemExpress. The nucleolin inhibitor AS1411 and a negative control (CRO) were kindly provided by Professor Richard G. Hegele, University of Toronto.

### RSV Infection and Flow Cytometry Analysis of A549 Cells

A549 cells were treated with oligonucleotides or RSV fusion inhibitors for 2h prior to RSV-GFP infection. Cells were exposed to RSV at a multiplicity of infection (MOI) of 1, 0.1, 0.05, or 0.01. Cells were incubated with the virus for 2 h at 37°C 5% CO_2_ to allow entry and then washed in PBS. The cells were supplied with new cell culture media and incubated for 37°C and 5% CO_2_. At indicated time points post infection (p.i.), the cells were washed and trypsinized prior to staining with a LIVE/DEAD® Fixable near-IR Dead Cell Stain Kit (Life Technologies). The data acquisitions were obtained on a FACSVerse (BD) and all analyses was performed with FlowJo software (Tree Star).

### Respiratory Syncytial Virus Cell Surface Binding Assay

A flow cytometry-based RSV cell surface binding assay was performed as previously described (9) with minor alterations. Briefly, cells were detached with 2mM EDTA, chilled on ice and pre-incubated with ssON (1 µM), AS1411 (25 µM), or CRO (25 µM) for 30 min at 4°C followed by incubation with RSV (MOI 2) for 1h at 4°C to prevent entry of the virus. The cells were thereafter extensively washed, prior to fixation in 4% Formaldehyde and treatment with 0.01% Triton X-100. The cells were stained with RSV anti-F antibody (1:200; Ab43812, Abcam) overnight at 4°C followed by staining with APC-conjugated rat anti-mouse IgG1 secondary antibody (1:100; Invitrogen) for 1h at RT. The data acquisitions were obtained using a FACSVerse (BD) and all analyses was performed with FlowJo software (Tree Star).

### Nucleolin Binding Assay

ssON’s interaction with nucleolin expressed on the surface of cells was assessed using a modified version of the flow cytometry-based cell surface binding assay (9). A549 cells were detached with 2mM EDTA, chilled on ice and pre-incubated with ssON (1 µM) for 30 min at 4°C, followed by incubation with anti-nucleolin antibody (Ab22758, Abcam) at different concentrations for 1h at 4°C and then washed. The binding of the anti-nucleolin antibody to the cell surface, was revealed by staining with Alexa 488-conjugated donkey anti-rabbit IgG secondary antibody (5 µg/ml; Invitrogen) for 1h at RT. Cells were then washed and the data acquisitions were obtained using a FACSVerse (BD) and all analyses were performed with FlowJo software (Tree Star).

### Ethics Statement

The *in vivo* studies were carried out in accordance with INRAE guidelines in compliance with European animal welfare regulation. The protocols were approved by the Animal Care and Use Committee at “Centre de Recherche de Jouy-en-Josas” (COMETHEA) under relevant institutional authorization (“Ministère de l’éducation nationale, de l’enseignement supérieur et de la recherche”), authorization number 201803211701483v2 (APAFIS#14660). All experimental procedures were performed in a biosafety level 2 facility.

### Mouse Experiments

Adult female BALB/c mice (> 8 weeks) were treated intranasally with ssON (50 µl; 12.5 µg or 6.25 µg) or PBS 6h prior to intranasal challenge of RSV-Luciferase (50 µl; 10^5^ pfu). Luminescence was measured at day 0, 2, 3, and 4 using an IVIS system (Xenogen Corp.) by anaesthetizing the mice using isoflurane and administering D-luciferin (Sigma) intranasally 5 min before capturing photon emission. The “Living Image” software (version 4.0. Caliper Life Sciences) was used to quantify the Luciferase activity. Bioluminescence images were acquired for 1 min with f/stop of 1 and binning of 8. Digital false-color photon emission images of mice were generated, and photons were counted within a constant region of interest corresponding to the nose or the lungs area. Photon emissions were measured as radiance in photons per second per square centimeter per steradian (p/s/cm2/sr). ssON was administered a second time on day 2 *via* intranasal administration (50 µl; 12.5 µg or 6.25 µg). The mice were anesthetized with isoflurane or ketamine/xylazine and euthanized on day 4 by cervical dislocation. Twelve mice were analysed in each group.

### Hematoxylin and Eosin Staining

Frozen OCT-embedded mouse lung (left lobe) tissue was cut into 12 μM sections using a Cryostat (Leica), transferred onto SuperFrost slides (ThermoFischer), and fixed with ice-cold acetone. H&E staining was then performed according to standard histopathological protocols. Briefly, sections were stained for 30 seconds in hematoxylin (Gill II, Roth) and dipped once in eosin Y (1% aqueous, Surgipath). Sections were then dehydrated using 70% and 100% ethanol, submerged in xylene, and mounted using DPX mounting medium (Sigma Aldrich).

### RNA Extraction and RT-qPCR

Once the animals were euthanized, the lungs were perfused through the right ventricle and then immediately collected and placed in RNALater (Qiagen). The RNA was extracted from the accessory lung lobe, or from another right lobe, from mice treated with either PBS, ssON (6.25 µg), RSV, or RSV + ssON (6.25 µg) using the RNeasy® Plus Mini kit (Qiagen) according to manufacturer’s recommendations. Briefly, the tissue was homogenized in RLT buffer supplemented with β-mercaptoethanol using an mTube (Miltenyi biotech) with the gentleMACS dissociator (Miltenyi Biotech). The lysates were transferred to gDNAse eliminator spin columns prior to extensive washing followed by RNA elution. The obtained concentrations of RNA were around 400±200 ng/μl and the obtained value for A260/280 were around 2.06±0.07 and A260/230 were around 2.1±0.1, which indicate a pure RNA eluate with no solvent contamination. cDNA was produced using SuperScript™ III Reverse Transcriptase (Invitrogen) according to manufacturer’s instructions. Real-time PCR reactions were prepared using the QuantiFast SYBR green kit (Qiagen) according to manufactures recommendations. The relative gene expression was measured using primers for *Gapdh* (Qiagen), *Stat1* (BioRad), *Stat2* (Qiagen), *Ccl2* (Qiagen), *Cxcl10* (Qiagen), *Tnf* (Qiagen), *Ccl4* (Qiagen), and *Ifitm1* (Qiagen). Following the initial denaturation step at 95°C for 5 min, 45 cycles constituting 5 s at 95°C, 30 s at 60°C and 1 min at 65°C were run on a Light cycler 480 II (Roche). Melting point analysis confirmed that all PCR reactions yielded only a single product species and gene expression was calculated with the delta-delta CT method using *Gapdh* as a reference gene. All data are presented as fold change of the respective negative control.

### Nanostring Analysis

The gene expression profile in 44 samples were analysed using the nCounter Mouse Immunology Panel (Nanostring Technologies, Seattle, QA). The immunology panel profiles 561 genes; 547 immunology-related genes and 14 internal reference controls. Each sample contained 50ng of total RNA which were prepared according to manufacture’s instructions and analysed with the automated nCounter Prep Station (Nanostring Technologies). For normalization and quality control the analysis software nSolver v3.0.22 (Nanostring Technologies) was used. A detection threshold was set using the mean plus 3 standard deviations of the negative controls. For differential gene expression, raw values were imported into R (v 3.6.0), and pairwise comparisons between conditions were performed with DEseq2 (v 1.26.0), after normalizing the immune gene expression values by specifying the internal reference genes to the control genes parameter within the *estimate Size Factors* function (36). Genes with an adjusted p-value below 0.05 were marked as differentially expressed in the MA-plots and for the volcano plots, genes with an adjusted p-value below 0.01 were marked as differentially expressed. The principle component analysis was conducted by the *prcomp* function in base R, after regularized log transformation with DESeq2. DESeq2 analysis was used to normalize the immune gene expression to the internal reference genes and the top 100 most variable genes after rlog transformation were selected for heatmap visualization of z-scores using the *scale* function in base R.

### Statistics

All data were analyzed using the GraphPad Prism software version 6.07. The different RSV inhibitors (TMC353121 and Presatovir) and ssON were compared with the RSV control by using the Kruskal-Wallis one-way ANOVA test with Dunns multiple comparison. The non-parametric Mann-Whitney test was used for all other *in vitro* experiments by comparing the different treatments to the RSV or nucleolin controls. The statistical significance for all *in vivo* experiments were measured using the unpaired t-test with Welch’s correction comparing each treatment to the RSV control. The Kruskal-Wallis one-way ANOVA test with Dunns multiple comparison was used for the RT-qPCR experiments by comparing the different treatments to the PBS and ssON controls. *P*-value: *P* > 0.05; * *P* ≤ 0.05; ** *P* ≤ 0.01; *** *P* ≤ 0.001; **** *P* ≤ 0.0001. Lack of significance was not depicted in the figures if no significance were observed between treatments. All data are presented as the mean ± standard error of the mean (SEM). The number of individuals and repeated experiments are stated in each figure legend.

## Data Availability Statement

The raw data supporting the conclusions of this article will be made available by the authors, without undue reservation.

## Ethics Statement

The animal study was reviewed and approved by the Animal Care and Use Committee at “Centre de Recherche de Jouy-en-Josas” (COMETHEA) under relevant institutional authorization (“Ministère de l’éducation nationale, de l’enseignement supérieur et de la recherche”), authorization number 201803211701483v2 (APAFIS#14660).

## Author Contributions

SP and A-LS designed and performed the experiments, analyzed the data, and wrote the manuscript. AD, LS, RG, and MG performed the *in vivo* experiments. JB and AB performed and analysed the nanostring experiments. JL and MW-H supervised nanostring analyses. AD and CR performed and analysed the RT-qPCRs. RG analysed the *in vivo* data. M-AR-W and J-FE provided reagents. UP and PM provided reagents and critical feedback. All authors read and reviewed the manuscript. All authors contributed to the article and approved the submitted version.

## Funding

This work was supported by the Swedish Research Council https://www.vr.se/ (521–2014–6718) awarded to A-LS. This study was in part funded by the European Union's Horizon 2020 in response to the corona virus outbreak for research and innovation action under grant agreement No. 101003555.

## Conflict of Interest

AD and A-LS are shareholders of TIRmed Pharma in possession of intellectual properties related to ssON. A-LS is CEO of TIRmed Pharma.

The remaining authors declare that the research was conducted in the absence of any commercial or financial relationships that could be construed as a potential conflict of interest.
