# Supplementary material for: Single-Stranded Oligonucleotide-Mediated Inhibition of Respiratory Syncytial Virus Infection
Source: Front Immunol. 2020 Dec 8;11:580547. doi: 10.3389/fimmu.2020.580547 (PMC7752805; doi:10.3389/fimmu.2020.580547)
Supplement: Supplementary file 1 [file DataSheet_1.pdf]

SUPPLEMENTARY FIGURES

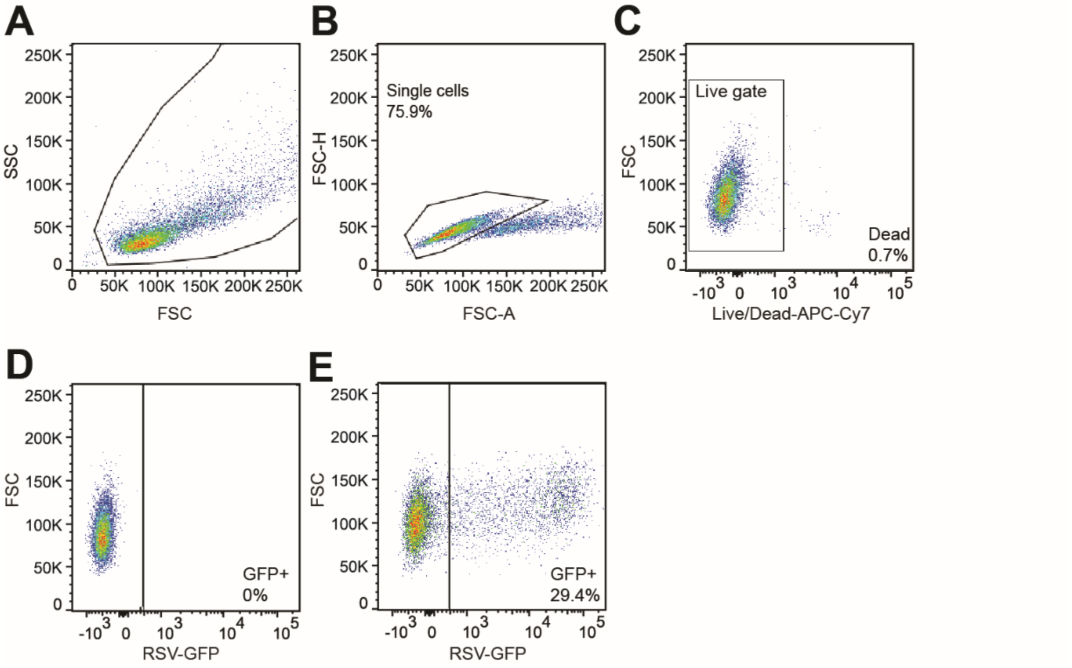

Supplementary Figure 1. Flow cytometry gating strategy for A549 cells
(**A**) A549 cells were distinguished from debris using forward-scattered light (FSC) and side-scattered light (SSC). (**B**) Single cells were then selected using FSC-Area versus FSC-Height and subsequently, (**C**) dead cells were excluded using Live/Dead fixable near-IR dead cell stain kit (APC-Cy7). (**D**) The proportion of infected cells was determined using a GFP gate. (**E**) Example of the GFP gate in an RSV infected sample.

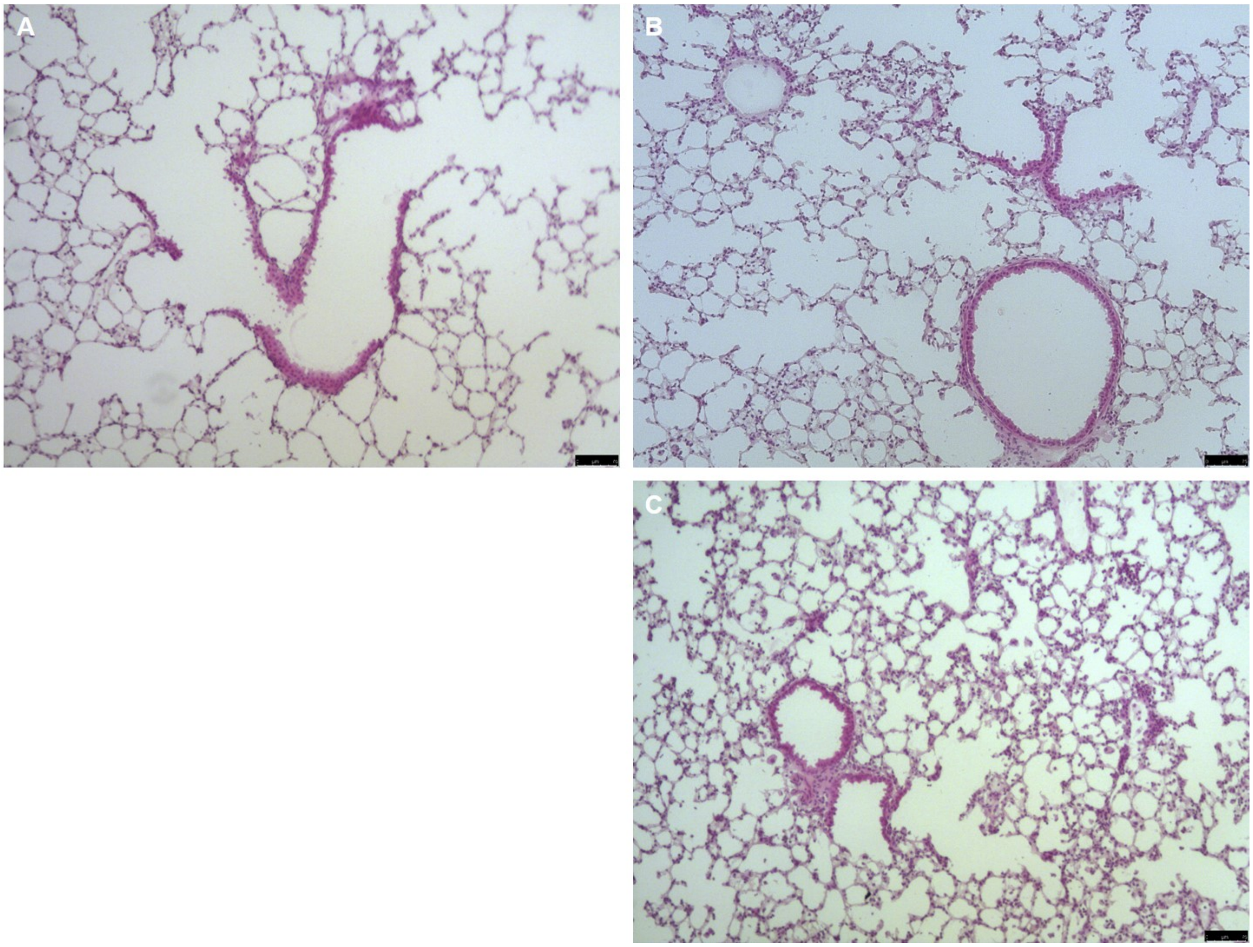

Supplementary Figure 2. RSV infection alone or RSV infection in combination with ssON treatment did not affect the overall pathology in murine lungs

Hematoxylin and eosin staining of lungs from mice treated with (**A**) PBS, (**B**) RSV or (**C**) RSV and ssON (6.25μg), 4 days post infection. Representative pictures for each treatment group are shown at a 10X magnification. Scale bars = 75μm.

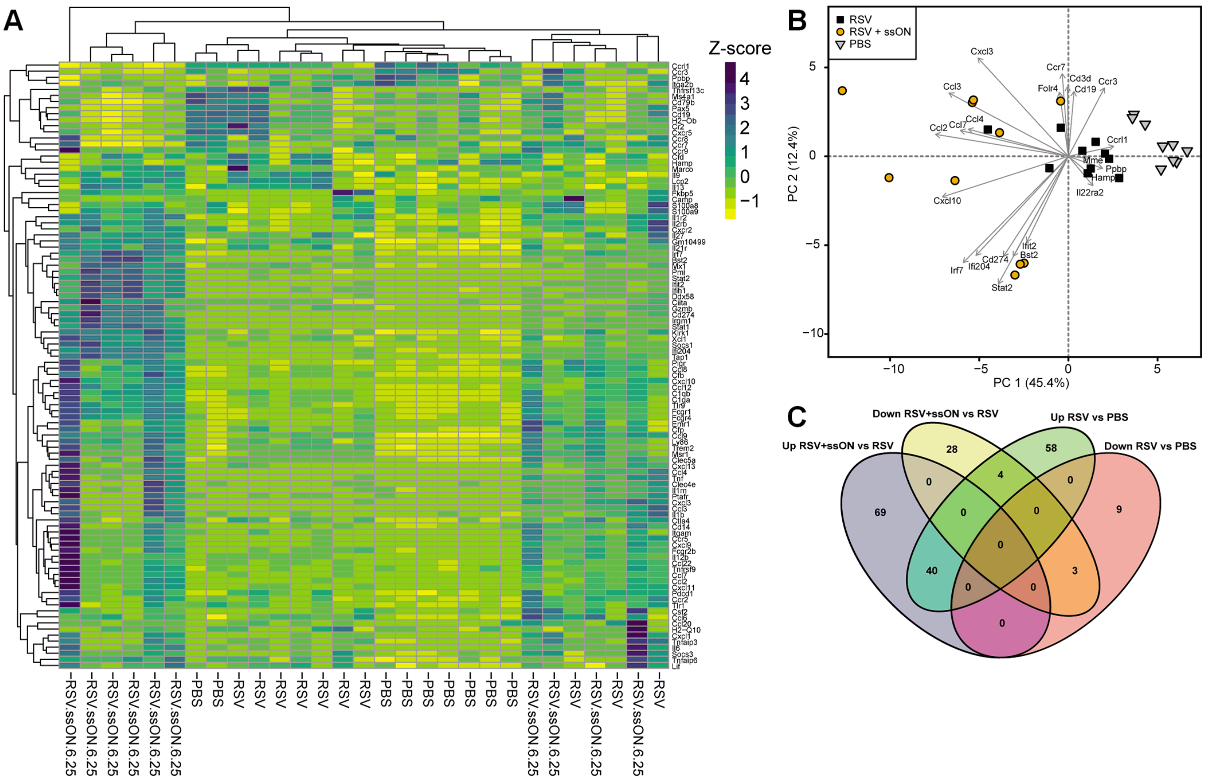

Supplementary Figure 3. RSV infection alone and RSV infection in combination with ssON treatment results in a differential immune profile in the lungs of mice

RNA was extracted from the right lung lobes of mice treated with PBS, infected with RSV or from RSV infected mice treated with ssON (6.25µg) 4 days post infection and analyzed using Nanostring technology. Data consist of 9-10 mice per treatment from two independent experiments. (**A**) DESeq2 analysis was used to normalize the immune gene expression to the internal reference genes and the top 100 most variable genes after rlog transformation were selected for heatmap visualization of z-scores using the *scale* function in base R. (**B**) Principle component analysis (PCA) plot of sample after immune gene expression-based RNA profiling. DESeq2 analysis was used to normalize the immune gene expression to the internal reference genes and a PCA was conducted after regularized log transformation of the data. The top 10 genes based on variable loadings for the first two principal components are displayed in the graph. (**C**) Venn diagram after differential gene expression comparisons of immune gene expression profiles. DESeq2 was used to normalize the immune gene expression to the internal reference genes and to conduct differential gene expression analysis for two independent comparisons: (i) RSV vs PBS and (ii) RSV + ssON vs RSV. For each comparison, genes with adjusted p-value < 0.05 were extracted and used for creation of the Venn diagram.

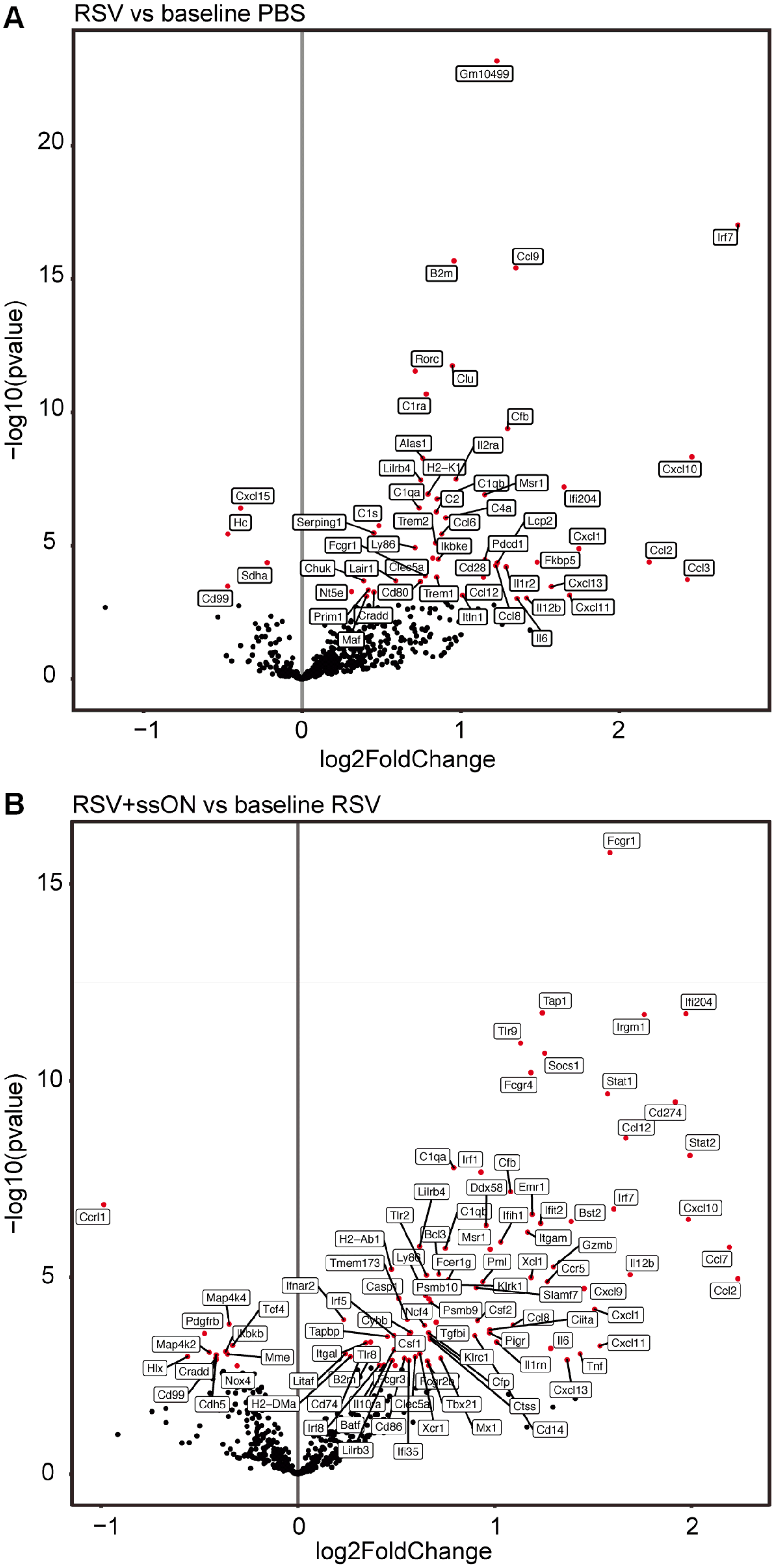
A
RSV vs baseline PBS
-log10(pvalue)
log2FoldChange
-1
0
1
2
0
5
10
15
20
Gm10499
Irf7
Ccl9
B2m
Rorc
Clu
C1ra
Cfb
Alas1
Il2ra
Lilrb4
H2-K1
C1qb
Msr1
C1qa
C2
C4a
Ifi204
Cxcl10
Cxcl15
Hc
C1s
Serping1
Trem2
Ccl6
Lcp2
Cxcl1
Fcgr1
Ly86
Ikbke
Pdcd1
Fkbp5
Ccl2
Ccl3
Chuk
Lair1
Clec5a
Cd28
Sdha
Il1r2
Cxcl13
Nt5e
Cd80
Trem1
Ccl12
Il12b
Cxcl11
Cd99
Prim1
Cradd
Itln1
Ccl8
Il6
Maf
B
RSV+ssON vs baseline RSV
-log10(pvalue)
log2FoldChange
-1
0
1
2
0
5
10
15
Fcgr1
Tap1
Ifi204
Tlr9
Irgm1
Socs1
Fcgr4
Stat1
Cd274
Ccl12
Stat2
C1qa
Irf1
Cfb
Ddx58
Emr1
Lilrb4
Irf7
C1qb
Ifit2
Tlr2
Ifih1
Bst2
Cxcl10
Ccrl1
Bcl3
H2-Ab1
Msr1
Itgam
Gzmb
Ly86
Ccl7
Tmem173
Fcer1g
Pml
Xcl1
Il12b
Ccr5
Casp1
Psmb10
Klrk1
Slamf7
Cxcl9
Ccl2
Ifnar2
Map4k4
Irf5
Tcf4
Ncf4
Psmb9
Csf2
Cybb
Ccl8
Ciita
Cxcl1
Pdgfrb
Ikbkb
Tapbp
Tgfbi
Pigr
Il6
Cxcl11
Map4k2
Csf1
Mme
Itgal
Tlr8
Klrc1
Il1rn
Tnf
Hlx
Cradd
Nox4
Litaf
B2m
Fcgr3
Fcgr2b
Cfp
Cxcl13
Cd99
Cdh5
H2-DMa
Cd74
Il10ra
Clec5a
Tbx21
Ctss
Irf8
Batf
Cd86
Xcr1
Mx1
Cd14
Lilrb3
Ifi35

Supplementary Figure 4. ssON treatment *in vivo* induces a differential immune profile in the lungs of RSV infected mice

RNA was extracted from the right lung lobes of mice treated with PBS, infected with RSV or from RSV infected mice treated with ssON (6.5µg) 4 days post infection and analyzed using Nanostring technology. Data consist of 9-10 mice per treatment from two independent experiments. DESseq2 analysis was used to normalize the immune gene expression to the internal reference genes. Genes with an adjusted p-value below 0.01 are marked with red color. Volcano plots showing the expression profile of immune genes in (**A**) RSV infected mice compared to PBS treated mice and (**B**) ssON treated RSV infected mice compared to RSV infected mice.

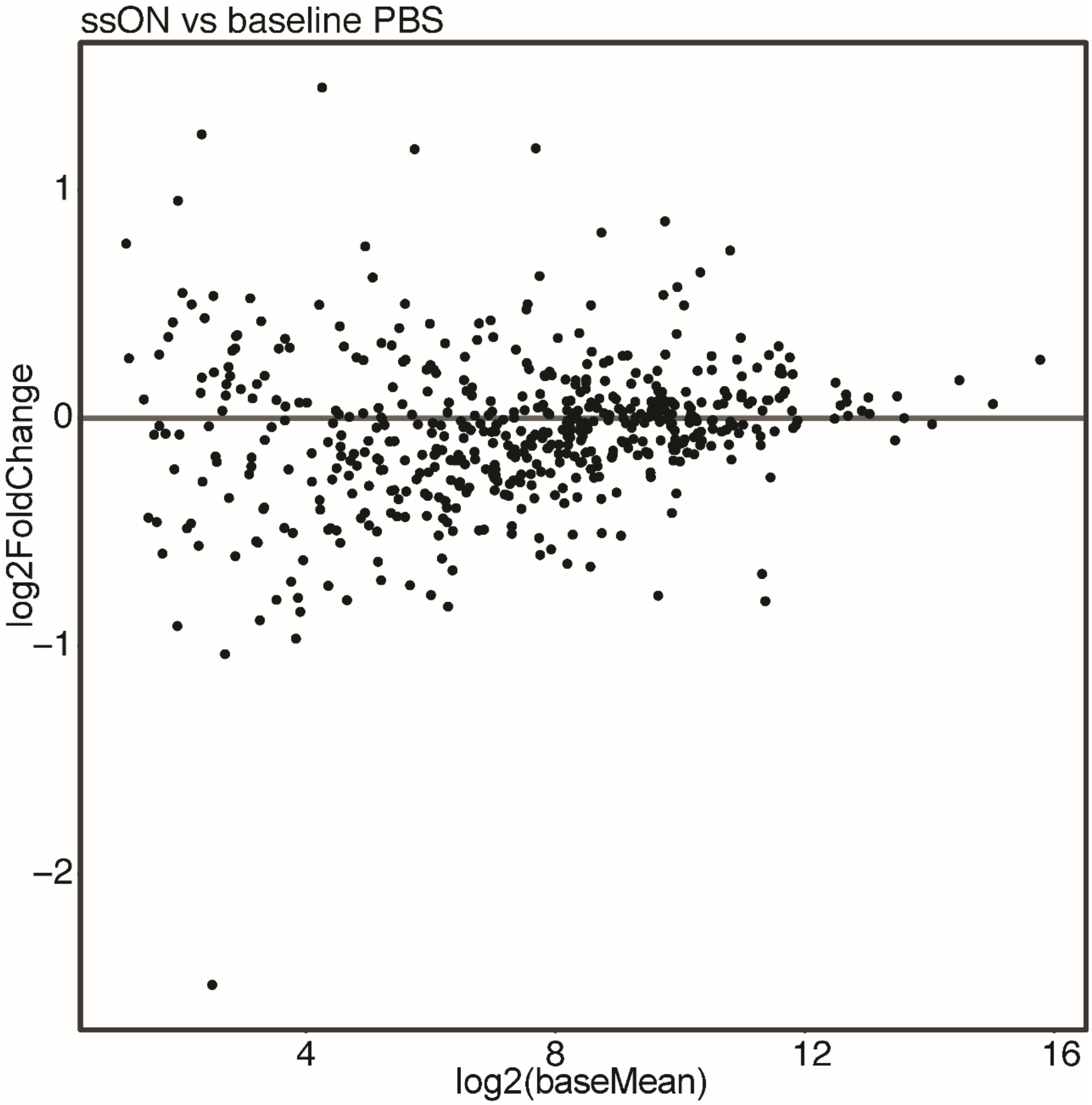

Supplementary Figure 5. ssON treatment in the absence of RSV infection does not induce any significant upregulation or downregulation of ISGs

RNA was extracted from the right lobes of mice treated with PBS or ssON (6.25µg) 4 days post treatment and analyzed using Nanostring technology. Data consist of 5-10 mice per treatment from two independent experiments. DEseq2 analysis was used to normalize the immune gene expression to the internal references genes. MA-plots showing the expression profile in log2 fold change of immune genes in ssON treated mice compared to PBS treated mice.

Supplementary Table 1. List of ISG genes

Table show log2foldchange of the ISGs included in nanostring analysis. Significantly upregulated genes marked in bold for each treatment.

**Gene**	**RSV* infection compared to PBS^ treatment**	**ssON treatment of RSV infection¨ compared to RSV* infection**
B2m	**0.96**	**0.37**
Bcl3	0.31	**0.71**
Bst2	**0.90**	**1.39**
C1s	**0.48**	0.03
Casp1	0.23	**0.51**
Ccl19	**0.84**	-0.26
Ccl2	**2.19**	**2.23**
Ccl4	**1.78**	1.29
Ccl5	0.22	0.31
Ccl8	**1.22**	**1.09**
Cd163	0.32	-0.18
Cd247	0.05	**0.02**
Cd69	0.03	0.33
Cd74	-0.08	**0.33**
Cd80	**0.74**	**0.46**
Cd9	0.04	-0.01
Cdkn1a	0.22	0.04
Ceacam1	0.10	0.01
Cfb	**1.29**	**1.07**
Cx3cl1	-0.29	-0.39
Cxcl10	**2.46**	**1.98**
Cxcl11	**1.69**	**1.53**
Cxcl9	**1.11**	**1.45**
Ddx58	0.35	**0.95**

Defb1	0.96	0.17
Fkbp5	**1.48**	-0.31
Gzmb	**0.87**	**1.29**
Ifi35	0.26	**0.56**
Ifih1	0.36	**1.03**
Ifit2	0.54	**1.23**
Ifitm1	0.30	**0.42**
Ifngr1	0.16	-0.21
Il15	0.31	0.08
Il15ra	0.42	-0.03
Il17rb	0.23	-0.18
Il1rn	0.62	**1.00**
Il6st	-0.05	-0.15
Irf1	-0.08	**0.93**
Irf7	**2.75**	**1.60**
Msr1	**1.15**	**0.97**
Mx1	0.58	**0.72**
Myd88	**0.35**	**0.31**
Nfil3	0.07	0.17
Nod2	0.35	0.54
Pml	0.17	**0.94**
Psmb9	0.33	**0.67**
S100a8	0.31	-0.08
Serping1	**0.45**	-0.06
Smad3	-0.05	-0.22
Socs1	0.03	**1.25**
Stat1	0.30	**1.57**
Stat2	0.76	**1.99**
Stat3	0.13	0.14

Tagap	-0.09	0.10
Tap1	**0.55**	**1.24**
Tgfb1	-0.05	0.05
Tlr3	0.15	0.30
Tnfaip3	**0.81**	0.43
Tnfaip6	0.58	0.58
Tnfrsf13b	0.02	0.28
Tnfsf10	-0.27	0.46

*RSV n=10, RNA collected 4 days post-infection

^PBS n=9

¨RSV and ssON (6.25µg) n=10, RNA collected 4 days post-infection
